# Accurate Prediction of the Dynamical Changes within the Second PDZ Domain of PTP1e

**DOI:** 10.1371/journal.pcbi.1002794

**Published:** 2012-11-29

**Authors:** Elisa Cilia, Geerten W. Vuister, Tom Lenaerts

**Affiliations:** 1 MLG, Département d'Informatique, Université Libre de Bruxelles, Brussels, Belgium; 2 Department of Biochemistry, University of Leicester, Leicester, United Kingdom; 3 AI-lab, Vakgroep Computerwetenschappen, Vrije Universiteit Brussel, Brussels, Belgium; Institut Pasteur, France

## Abstract

Experimental NMR relaxation studies have shown that peptide binding induces dynamical changes at the side-chain level throughout the second PDZ domain of PTP1e, identifying as such the collection of residues involved in long-range communication. Even though different computational approaches have identified subsets of residues that were qualitatively comparable, no quantitative analysis of the accuracy of these predictions was thus far determined. Here, we show that our information theoretical method produces quantitatively better results with respect to the experimental data than some of these earlier methods. Moreover, it provides a global network perspective on the effect experienced by the different residues involved in the process. We also show that these predictions are consistent within both the human and mouse variants of this domain. Together, these results improve the understanding of intra-protein communication and allostery in PDZ domains, underlining at the same time the necessity of producing similar data sets for further validation of thses kinds of methods.

## Author Summary

Intra-protein communication has recently attracted an increasing interest from the scientific community, because of its important functional consequences: allostery and signalling. Unravelling how information is processed and transferred within a protein structure requires the study of the dynamical effects of, for instance, binding events, which may be captured experimentally by NMR relaxation experiments. Given the complexity of this experimental analysis, computational approaches, often based on molecular dynamics simulations, have been proposed for predicting these dynamical effects, using protein structural information as input. We examine here the accuracy of these predictors in the context of a well-studied domain, i.e. the second PSD95/Disc-large/ZO-1 domain (or PDZ domain) of PTP1e, and compare it to our approach that combines Monte-Carlo sampling of the conformational space of the side-chains and an information theoretical analysis. The results we discuss in this manuscript show clearly that the latter method provides very accurate predictions when compared to the experimental results, and has a better predictive quality compared to other computational approaches. The predictions, which are consistent between closely related structures, and the global network perspective provided by this approach, improve our understanding of intra-protein communication and allostery in these domains.

## Introduction

PDZ (PSD95/Disc-large/ZO-1) domains [1] are structural modules, consisting of about a hundred amino acids, common to many signaling proteins. They have been shown to not only serve as scaffolds for other proteins but also to possess particular dynamical properties [2], which are either induced by the direct interaction with a peptide [3], [4], [5] or indirectly by the interaction with neighboring domains [6], [7]. These properties demonstrate that specific forms of intra-protein communication are present within PDZ domains. Historically, such intra-protein communication is denoted as allostery when the communication serves a functional role [8]. Allostery traditionally has been explained using either the induced-fit model [9] or the more general applicable conformational equilibria model [10]. Whereas the first provides a mechanistic explanation, the latter does not require sequential mechanisms for the propagation of information and production of allosteric effects. Yet, both these classical models assumed large structural differences between the different macroscopic states of the protein, like for instance their bound and unbound forms. Recently, it has been argued that long-range intra-protein communication may originate from the changes in internal dynamics, without altering the average macroscopic conformation, providing as such information exchange only through entropic effects [11], [12], [13], [14], [15], [16].

In order to understand and predict the nature of such intra-protein communication, structural and dynamical data regarding proteins or domains in their major states are necessary. In this sense, the PDZ data gathered by Andrew Lee and colleagues [3], [17], [18] provide an important resource that allows fundamental questions, related to the role of dynamics in the information propagation throughout a protein, to be answered. Using NMR and crystallography data of the second PDZ (hPDZ2) domain of the human protein tyrosine phosphatase 1E (PTP1E), Lee and colleagues showed that i) binding a RA-GEF2 C-terminal peptide affects the methyl side-chain dynamics of a restricted set of residues within the domain [3], ii) mutating particular residues within that set alters peptide affinity [18], iii) interfering with these residues has long-range dynamical effects throughout the domain structure [18], and iv) all these changes are mostly related to side-chain equilibrium dynamics as opposed to backbone structural changes [17]. In addition they showed that side-chain dynamics are mostly conserved within the PDZ domain family [19], although not every PDZ domain incorporates the same dynamical changes [5]. This underlines the finding that the PDZ domain family can be divided into different functional categories related to their implicit communicative behavior. It is tempting to speculate that such a functional partitioning is sensible since each PDZ domain has its own particular role within its encompassing structural context.

The dynamical hPDZ2 data also provides a unique validation set for computational methods that aim to predict intra-domain information exchange from either structural or sequence-related information. Kong and Karplus [20] used a molecular dynamics approach, called the interaction correlation analysis, to determine the residues involved in transmitting binding information to other parts of the hPDZ2 domain. They identified a wide range of effects including two signaling pathways, linking residues in the binding pocket and the L1 loop to distal locations in the domain structure. Recently, Gerek and Ozkan [21] applied their perturbation response scanning method to also analyze the hPDZ2 domain, proposing effects for regions closely situated to the top of the binding site. These alternative regions differed from the family-wide regions identified for the PDZ domain family at large [22] and varied in their overlap with the experimental data of Lee and colleagues. As such, one can question the quality of the current predictions with respect to the experimental data produced so far.

Using the publically available structural data on hPDZ2 [23], [24], combined with the experimental results [3], [17], [18], [25], we determine here the quality of the previously published computational approaches and compare these to the results produced through an information theoretical approach that we proposed earlier [26]. We demonstrate that the previous predictions for hPDZ2 either suffer from a high false positive rate, or, even worse, are close to the results one obtains for a random predictor. We show that our information theoretical approach reaches higher levels of accuracy with respect to the experimental data. Additionally, we determine the main information exchange patterns from the network of dynamical changes and examine their relationship with the signaling pathways proposed in [20], [21]. Finally, we show that dynamical changes induced by binding a peptide to the mouse variant of this domain (mPDZ2), which has been shown to experience larger structural changes [4], [27], cover the same collection of residues as those identified for the human variant.

## Results

### Predicting dynamic changes in the set of methyl-group containing residues

**Figure 1:**
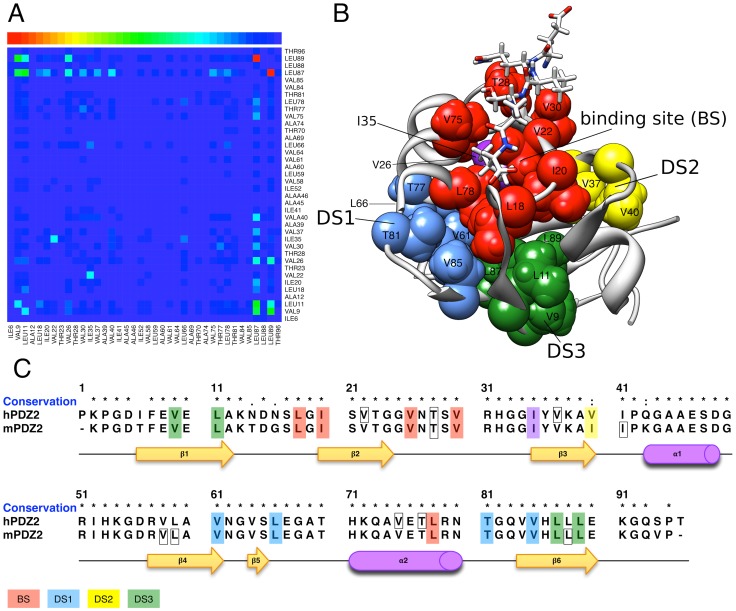
Predictions for methyl-group containing residues. A) The matrix of dynamical changes (heat map) for the methyl-group bearing residues, corresponding to a sub-matrix of the matrix in Figure S1 in Text S1. The matrix shows the absolute ΔMI, normalized between 0 and 1, colored from blue (0) to red (1) according to the color scale reported on top. B) Predictions mapped on the crystal structure (PDB-ID: 3LNY). Different colors are used for the different experimentally identified domain regions [3]. The red, blue and yellow residues correspond to the binding site (BS), the distal surface 1 (DS1), and the distal surface 2 (DS2) respectively. The residues in green are part of a newly identified distal region (DS3). I35 is highlighted in purple. The RA-GEF2 peptide is shown in stick representation. C) The predictions for the methyl-group containing residues highlighted in a sequence alignment of the two homologous domains (hPDZ2 and mPDZ2). The residues highlighted in red, blue and yellow correspond to the residues experimentally identified as affected by peptide binding in [3]. Residues composing the DS3 are highlighted in green and I35 in purple. Other predicted residues are squared in black.

To determine the residues throughout the hPDZ2 structure affected by peptide binding, we quantify the change in conformational coupling between residue side-chains within the major states of the domain, i.e. the bound and unbound states [26] (see Materials and Methods): In a first step, a Monte-Carlo sampling process was used to determine the conformational freedom of each residue, represented by a probability mass function over a fine-grain discrete set of side-chain conformations for each protein state. In a second step, these probability mass functions were used to calculate the mutual information (MI) between every residue pair, again for both protein states. A high MI value designates a high degree of coupling between the side-chain conformations of two residues, whereas a low value shows the opposite. Slight modifications with respect to the original method [26] were made in order to improve the calculation of the MI. The validity of this approach was recently confirmed in [28]. In the final step, the absolute differences between the MI values of the bound and unbound form were calculated (ΔMI), producing a matrix of mutual information changes (e.g. Figures 1A and Figure S1 in Text S1). Residue pairs displaying the higher absolute ΔMI value are involved in the dynamical changes induced by the peptide on the domain or protein structure.

Our analysis of these ΔMI values focuses first on the changes in the conformational couplings of methyl-group containing residues, as these results can directly be compared to the experimental data [3]. As such we extract from the matrix including all pairwise ΔMI values (see Figure S1 in Text S1) a sub-matrix (see Figure 1A) containing only the ΔMI values for all the pairs of methyl-group bearing residues of hPDZ2. In the next section we examine the prediction results for all residues. Note that in the current analysis we do not differentiate between negative and positive ΔMI values, focussing only on the magnitude of change and not the direction. In Figure S7 in Text S1, the raw (non-normalized) values corresponding to those shown in Figure 1A are reported.

Examining the sub-matrix in Figure 1A, one can see that the majority of the 38 methyl-group bearing residues are unaffected or only marginally affected by the binding event. Nevertheless, some residue pairs show ΔMI values significantly above background level, indicating particular peptide-induced conformational effects between residues. Using, as before, a clustering approach that extracts the network of most affected residue pairs (see Materials and Methods), we identified a set of dynamically affected residues, including residues located both at short and long distances from the binding pocket. The residues in this set are in agreement with those shown to be affected on the basis of experimental data [3] (see also Table 1). Figures 1B and 1C display our predictions mapped upon the hPDZ2 structure and sequence, showing the agreement with the known binding site (*BS*) residues L18, I20, V22, V26, V30 and L78 (predicting 6 out of 6 residues), as well as the majority of residues in the two distal surfaces identified in [3]: DS1 with 4 out of 6 residues identified (V61, L66, T81, V85), and DS2 with 1 identified residue out of 2 (V40).

**Table 1:** Summary of the results of the different predictors for the methyl-group bearing residues compared in Figure 3.

Method	Description	Predictions	tot. num.	TP	TN	FP	FN	AUC (Fig. 3A)	AUC (Fig. 3B)	TPR	FPR
Fuentes et al 2004	Experimental measurements	L18, I20, V22, V26, V30, A39, V40, V61, V64, L66, A69, L78, T81, V85	14	n. a.	n. a.	n. a.	n. a.	n. a.	n. a.	n. a.	n. a.
Kong and Karplus 2009	Molecular Dynamics (MD) approach	A12, L18, V26, I41, A45, A46, V58, L59, L66, A69, A74, L78, T81, L89	14	6	6	5	8	n. a.	n. a.	0.43	0.45
Gerek and Ozkan 2011	Perturbation Response Scanning method	L11, L18, I20, V22, T23, I35, V37, A39, V40, I41, A45, A46, V58, L59, A60, V61, L66, A69, A74, V75, T77, L78, T81, V85, L87	26	11	3	8	3	n. a.	n. a.	0.79	0.73
Predictions using METHYL data (Figure 1A)	Information theoretical approach applied to the methyl containing residues in the domain structure	V9, L11, L18, I20, V22, V26, T28, V30, I35, V37, V40, V61, L66, V75, T77, L78, T81, V85, L87, L89	20	11	9	2	3	0.74	0.81	0.79	0.18
Predictions using ALL data (Figure S1 in Text S1)	Information theoretical approach applied to all the residues in the domain structure	V9, L11, L18, I20, V22, V26, T28, V30, I35, V37, V40, L66, V75, T77, L78, T81, L87, L89	18	9	9	2	5	0.70	0.75	0.64	0.18
between centrality (bound)	Betweenness centrality measure (Del Sol, 2006) applied to the hPDZ2 bound form	L11, L18, I20, V22, V26, T28, I35, V37, V40, A46, I52, V58, L59, V61, L66, L78, V85, L87, L88, L89	20	9	6	5	5	0.54	0.59	0.45	0.64
between centrality (unbound)	Betweenness centrality measure (Del Sol, 2006) applied to the hPDZ2 unbound form	L11, L18, I20, V22, V26, T28, I35, V37, V40, A46, I52, V58, L59, V61, L66, A74, L78, V85, L87, L88, L89	21	9	6	5	5	0.56	0.55	0.45	0.64

For each method we report a short description and the corresponding set of predictions for hPDZ2 (columns 2 and 3). The Area Under the ROC Curves (AUC) calculated for the methods in Figure 3A and 3B is reported in columns 9 and 10, and the TPR and FPR of the best predictor selected from the ROC curve in Figure 3A are reported in columns 11 and 12. The TRP and FPR can be computed starting from the number of true positives (TP), true negatives (TN), false positives (FP) and false negatives (FN) in the columns from 5 to 8.

Interestingly, additional methyl-group containing residues are identified by our method, located in either BS (T28, V75), DS1 (T77) or DS2 (V37) (see Figure 1C). Others are located in a completely different domain region (V9, L11, L87, L89), which lies at the opposite of the binding cavity and which we propose to call distal surface 3 (DS3) corresponding to the residues coloured in green in Figure 1B and Figure 1C. Our method also predicts I35 to be affected by peptide binding. This residue, which is highlighted in purple in Figures 1B and 1C, is buried in the core of the domain and directly interacts with the BS residues V22 and L78 (in red) and with residues L66 and A69 in DS1 (in blue), therefore acting as a potential connector between these regions within hPDZ2.

### Identifying all dynamical changes in the hPDZ2 domain

Together with the set of methyl-group containing residues, our information theoretical approach identifies at the same time a number of non-methyl-group containing residues, which may have an important role in the intra-protein communication of hPDZ2. For example, residue H71, which has been extensively explored in the context of the evolutionary conserved network of energetically-coupled residues [29], displays ΔMI values comparable to L87 and L89. Using the same clustering method as before (see Material and Methods) we extracted the complete set (including both methyl-group bearing residues and non-methyl-group bearing residues) of affected residues from the ΔMI values in Figure S1 in Text S1, which we refer to as the *informative group*. This informative group covers the strongest dynamical effects induced by peptide binding over the entire domain.

**Figure 2:**
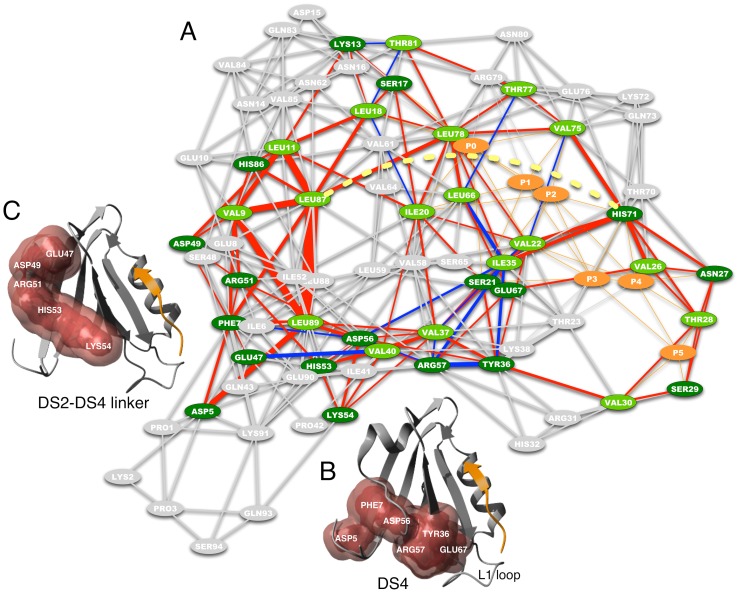
Network of short-range dynamical changes in hPDZ2. A) Residues highlighted in green are predicted on the basis of the complete matrix of changes in MI (light green for the methyl-group bearing side-chains and dark green for the others). Red edges represent an increase in MI, while blue edges represent a decrease in MI. The thickness of the edges represents the magnitude of change. Peptide residues and their contacts with the domain residues are highlighted in orange and the links connecting the peptide residues with the structure are not weighted. Yellow-dotted line illustrates an example of a long-range dynamical effect between LEU87 and HIS71. The network visualization follows the organic layout as implemented in Cytoscape [44]. B) The non-methyl bearing residues composing the continuous surface DS4, are highlighted in red on the ribbon structure of hPDZ2. C) The non-methyl bearing residues composing the continuous surface that links the two distal surfaces DS2 and DS4, are highlighted in red on the ribbon structure of hPDZ2.

To clearly visualize the informative group, we created a network of residue interactions including only those pairwise local interactions that have non-random ΔMI (see Materials and Methods) from the matrix of all dynamical changes (see Figure S1 in Text S1) and the crystal structures of hPDZ2 [17]. Figure 2A shows the resulting network for hPDZ2. On this network, we highlighted, using light (for methyl-group bearing residues) and dark (for non-methyl-group bearing residues) green colors (see Figure 2A), all the residues belonging to the informative group. The width and color of the edges indicate the strength and direction (increase or decrease) of the ΔMI value between the pairs of residues, using the ΔMI data similar to that shown in Figure S7 in Text S1. As expected, this weighted network shows that the majority of the green-highlighted residues become more tightly coupled (red edges) in response to the binding event, while fewer of them experience a relaxation (blue edges) in their conformational coupling. Of all residues, I35 experiences the largest number of decreases in conformational couplings with its structurally neighboring residues. At the same time, its conformational coupling with H71 and a number of other residues increases as a consequence of the binding between the RA-GEF2 peptide and hPDZ2. It should be noted that, in order to improve the readability, Figure 2A does not visualize the long-range ΔMI values present in Figure S1 in Text S1, such as the change in coupling between H71 and L87.

The clustering analysis on the ΔMI matrix visualized in Figure S1 in Text S1 added eighteen non-methyl-group bearing (dark green nodes in Figure 2A) residues to the ones discussed in the previous section (light green nodes in Figure 2A): A group of six connected residues (D5, F7, Y36, D56, R57, E67) comprises a region of the hPDZ2 domain that extends approximately perpendicularly to the binding groove, close to the L1 loop (Figure 2B). We subsequently refer to this region as distal surface 4 (DS4). These residues, together with H71 and the DS3 residues, can also be easily identified from the full matrix of dynamical changes (Figure S1 in Text S1). Other important residues are i) E47, D49, R51, H53 and K54 that extend DS4 towards helix α1 and the linker connecting this helix with the fourth β-strand (see DS2–DS4 linker in Figure 2C), and ii) K13, H86 and S17, S21, N27, S29 that enlarge DS1 and the BS region, respectively (see also network and alignment in Figure S6 in Text S1). Together with the other distal surfaces, the DS4 region creates a network that couples binding-pocket residues to residues in the α1–β4 linker and to DS3 residues located on β1 and β6 (see Figure 2A). This network combines dynamical effects located in the binding pocket, the domain core, and the bottom surface area of the domain.

### Assessing the predictive quality of the informative group

**Figure 3:**
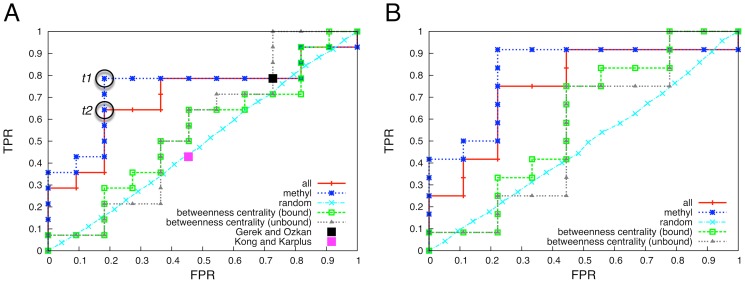
Quality assessment of the different predictors. A) ROC curves of the information theoretical approach considering either the dynamical changes in the methyl side-chain containing residues only (blue line) or dynamical changes for all the residue types (red line); the black square represents the performance of the Gerek and Ozkan predictor [21], the purple square represents the Kong and Karplus [20] predictor performance; grey and green lines represent the performance of a predictor ranking residues according to their betweenness centrality in the network derived from physical contacts using, respectively, the apo and the bound crystal structures. The two encircled points *t1* and *t2* correspond to the best performing predictors. B) The same as A, yet without the alanine residues, which were needed in the previous case for a fair comparison with the other approaches. We plot again the ROC curves of our approach compared to the baseline predictors in this setting.

To assess the predictive quality of our results, we computed their Receiver Operating Characteristics (ROC) curves (see Materials and Methods) and compared these to the curves produced by two null-models. Calculation of the ROC curves requires classification of the methyl-group containing residues as either *positives* (*P*) or *negatives* (*N*) on the basis of the experimentally determined dynamical data reported in [3], [18]. We excluded the residues for which the data are completely or partially missing from the quality analysis. On the basis of the classification (see Table S1 in Text S1), both the False Positive Rate (FPR) and True Positive Rate (TPR) levels were computed in response to a varying threshold *t*, required by the clustering method that we used to identify the informative group (see Materials and Methods). A high value of *t* limits the informative group to a few residues. Reducing the *t* value adds more and more elements until all residues are included in the set. In Table S3 in Text S1 we show the order in which residues become included in the informative group while changing *t*. Each change in *t* corresponds to a point in the ROC curves shown in Figure 3A, starting from the matrix of all residues changes (Figure S1 in Text S1) in red, and starting from the sub-matrix of methyl-group containing residues (Figure 1A) in blue. The quality of our predictor is compared to two null-models: i) a random predictor (light blue) and a predictor that scores the residues according to their betweenness-centrality (green and grey) computed from the network of residue contacts as derived from the X-ray structures of hPDZ2 (see Materials and Methods and Text S1).

Our predictions deliver significantly better results in terms of the area under the ROC curves (AUC), when compared to the curves derived for both the random and betweenness-centrality models (see Table 1). In case of methyl side-chain only analysis (Figure 3A), our predictor realizes an AUC of 0.74, compared to the 0.54–0.56 values of the betweenness-centrality predictor.

The difference between the all-residue and methyl-residue informative groups at low levels of FPR and TPR originates from the higher rank given by the first predictor to true negative residues T77 and I35 (see Table S3 in Text S1). This suggests that these two residues experience high ΔMI with non-methyl side-chain residues. Indeed, as one can see in Figure 2A, residue I35 shows significant side-chain effects with a large number of non-methyl-group residues. Conversely, I20 and V40 are ranked lower within the all-residue informative group (see Table S3 in Text S1), suggesting that these experience quite strong ΔMI with other methyl side-chain residues and smaller ΔMI with the non-methyl ones.

In order to understand the quality of our predictions, we also examined the accuracy of the two recently published methods [21] and [20] that also produced predictions for hPDZ2. We computed the FPR and TPR scores of the two methods based on the data presented in the two manuscripts (see Table 1). In Figure 3A the resulting quality assessments are shown as black and purple squares for the methods described in [21] and [20], respectively. The quality of the Kong and Karplus predictions is low and close to that of a random predictor, while the predictor proposed by Gerek and Ozkan yields a TPR value of 0.79, which is identical to the value obtained for the information theoretical approach presented here. However, the Gerak and Ozkan FPR value is 0.73, which makes their results qualitatively similar to the results of the random and betweenness-centrality models. As such both methods provide an overall poor accuracy, whereas our method seems to improve the accuracy considerably.

The ROC analysis also allows us to select a clustering threshold *t* corresponding to the best predictor, i.e. the one yielding the maximal TPR and minimal FPR value, which corresponds to the point of the ROC curve having the shorter Euclidean distance to the top-left corner of the plot in Figure 3A. The best predictor for the hPDZ2 data is obtained for *t_1_ = *0.023 (TPR = 0.79 and FPR = 0.18) for the methyl side-chain matrix of dynamical changes, and *t_2_ = *0.027 (TPR = 0.64 and FPR = 0.18) for the entire matrix of dynamical changes (see Figure 3A). These thresholds were used to produce the visualizations of the data displayed in Figures 1 and 2. Removing the conformationally invariant alanine residues from the ROC computation did not affect the optimal clustering thresholds.

### Incorporating the backbone differences in the predictions

**Figure 4:**
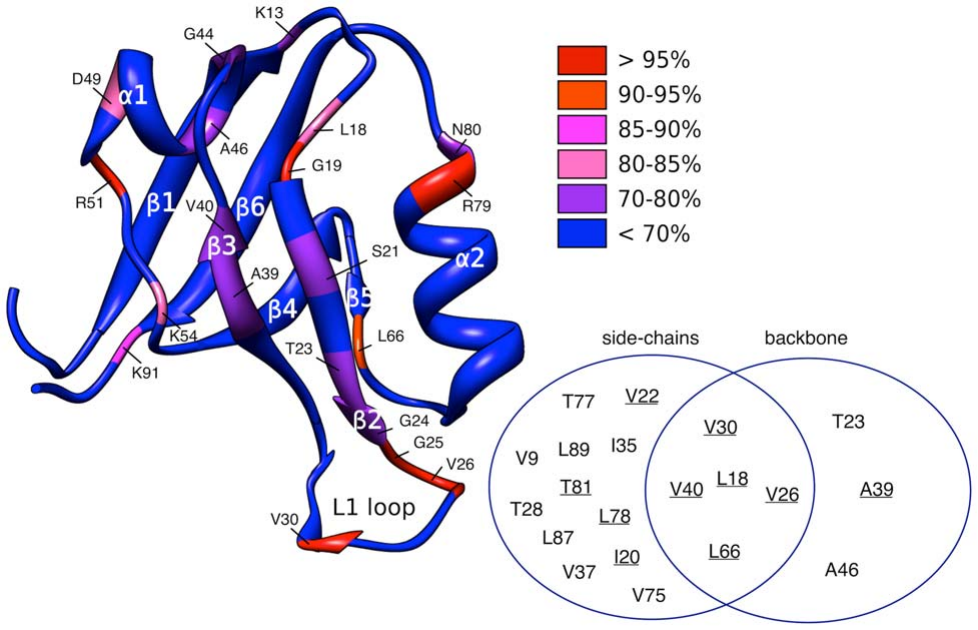
Changes in backbone flexibility as a result of peptide binding. The variations are mapped on the hPDZ2 crystal structure (PDB-ID: 3LNY). The different colors highlight the level of significance of the change, determined by z-scores. The Venn's diagram represents the predictions of methyl-containing residues obtained by our information theoretical approach (side-chains), compared to those obtained by analyzing the backbone variations (backbone). The underlined residues are those obtained from the experimental results in [3].

To improve the predictive performance, we investigated the possibility of exploiting backbone variation information derived from the available NMR ensembles of hPDZ2. We computed the residue-specific changes in backbone flexibility (see Materials and Methods) and displayed those onto the domain (see Figure 4). It is known from experimental studies that the hPDZ2 domain does not experience large structural changes [17], as also expressed by a RMSD of 0.4 Å between equivalent atoms of the crystal structures of the two states (ligand-free, 3LNX chain A, and ligand-bound, 3LNY). Similarly, variations from the NMR structures between the two states are below 0.55 Å, when excluding the flexible regions at the C- and N-termini.

Figure 4 highlights the residues that experience the most significant changes in backbone flexibility. As expected, the most affected regions are present in the binding pocket. Our results indicate that there is a clear change in variation in these regions relating most likely to a rigidification, in agreement with similar effects derived from NMR backbone relaxation experiments [3]. Some additional interesting regions also emerge from the data: residues K54, R51 D49, A46, and G44 within the α1 helix and the α1–β4 linker experience significant effects. A similar pattern of residues was identified previously for the mPDZ2 variant upon binding to the peptide of the human Fas receptor [27]. Figure 4 shows in a Venn diagram the difference in the predictions we obtained by considering the side-chain dynamics and the backbone variation information, separately. The underlined residues are those affected by peptide binding according to the NMR relaxation experiments.

We then tested the incorporation of the backbone variation information as a means to improve our approach. Upon combining this information with the earlier predictions, we obtained a slight improvement from AUC = 0.74 to AUC = 0.75, mainly resulted from the improved ranking of the alanine residues (e.g. A39), which cannot be considered significant. The ROC curve for this combined predictor is reported in Figure S3 in Text S1.

### Predicting the dynamical changes in mPDZ2

**Figure 5:**
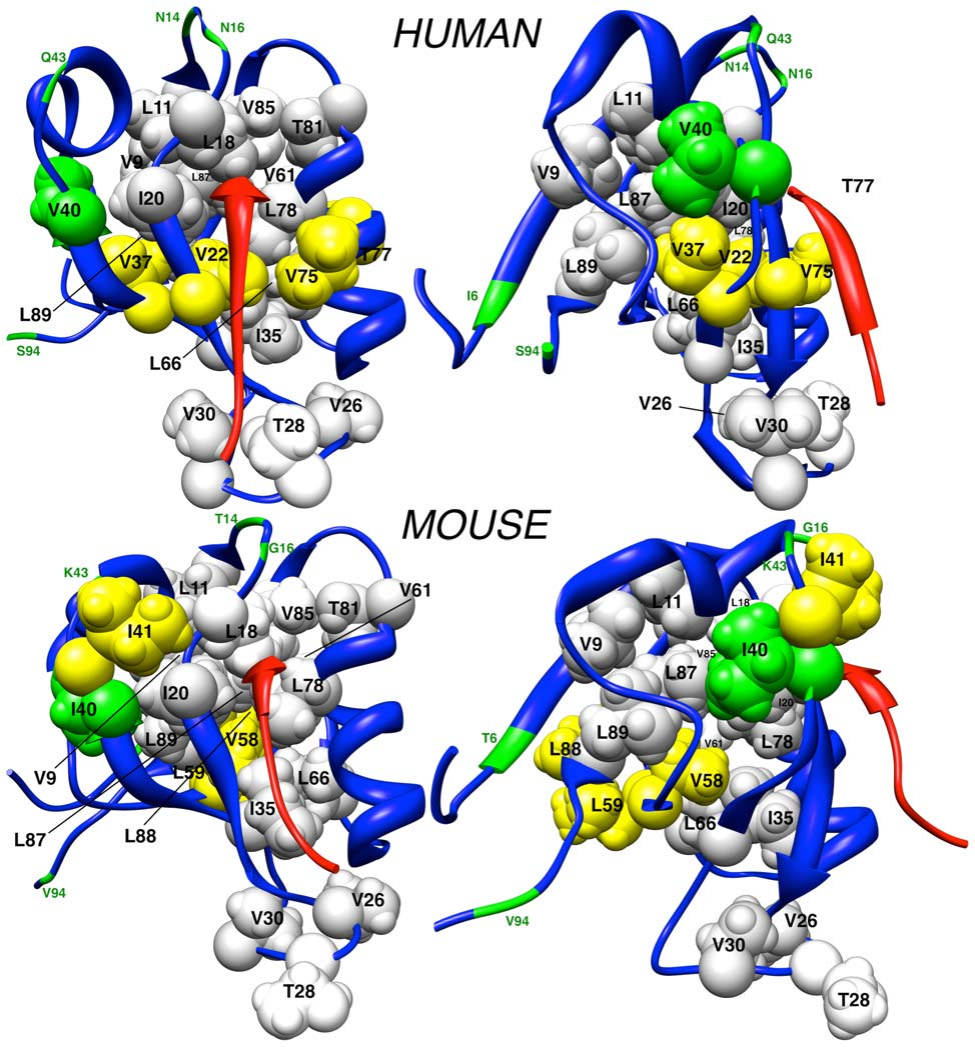
Similarities and differences in the predicted networks of dynamical changes in hPDZ2 and mPDZ2. Predictions related to the residues containing methyl-groups on the side-chain at threshold *t1 = *0.023. The picture shows two different domain orientations. The first orientation faces the binding groove; the second one, obtained by rotating the first one about 90 degrees to the right, shows the binding groove left side. Methyl-group bearing residues predicted for both the domains are shown in white, while differences in the predictions are highlighted in yellow. Amino acid differences between hPDZ2 and mPDZ2 are highlighted in green on the structure (see also the alignment in Figure S6 in Text S1).

Given the encouraging quality of our predictions on hPDZ2, we decided to analyze the equivalent PDZ domain of the mouse homolog PTP-BL (subsequently denoted as mPDZ2). This domain has a 94% sequence identity with hPDZ2, differing mostly in a few of amino acids along the β1–β2 and the β3-α1 linkers (see the alignment reported in Figure 1C and green residues in Figure 5). Nevertheless, NMR analysis has shown significant differences between these closely related domains, with mPDZ2 experiencing larger structural changes in the orientation of the second helix upon binding to the APC-derived peptide [4], as compared to the binding effects observed in hPDZ2. To understand the impact of these differences on the communicative mechanisms and to identify possible commonalities, we applied our information-theoretical approach also on the NMR ensembles of mPDZ2, producing a new matrix of dynamical changes (Figure S4 in Text S1) and the corresponding network (Figure S5 in Text S1). The clustering algorithm was used to extract the most affected residues from the matrix of mPDZ2 using the best threshold *t* as derived for the hPDZ domain (*vide ante*).

Barring inconsequential differences between the two matrices (Figure S1 and S4 in Text S1), the resulting informative groups for both hPDZ2 and mPDZ2 are quite similar (see Figure 1C for methyl-group containing residues only and the alignment in Figure S6 in Text S1 for all residues). The methyl side-chain residue predictions indicate that almost all binding pocket residues and distal group members (from DS1 to DS3) are shared between hPDZ2 and mPDZ2. By following the same numbering used for hPDZ2 according to the sequence alignment shown in Figure 1C, we show that also for mPDZ2, residue I35 is predicted as a relevant residue for the domain intra-communication, as is residue T28. Even though the data for hPDZ2-T28 are missing, mutation mPDZ-T28 affected binding affinity [4]. Our method also predicts for mPDZ2 the four residues comprising the DS3 region of hPDZ2. A pathway of methyl-methyl contacts that links L18 or L78 in the BS, to V85 and L87 was previously identified [4]. The same pathway is also present in our network of dynamical changes (Figure S5 in Text S1).

Comparison of the hPDZ2 and mPDZ2 networks (cf. Figures 2 and Figure S5 in Text S1) shows that they share in addition thirteen non-methyl bearing residues. The majority of these residues are those previously identified as DS4 (D5, F7, Y36, D56, R57), confirming the presence of this information exchange, as previously also identified in [20]. The other conserved predictions (see alignment in Figure S6 in Text S1) are: i) H71 in the binding site, ii) H53, K54 together with E47 and D49 in the α1 helix, which are part of the same linker region shown in Figure 1C, iii) K13 in the β1–β2 loop, iv) H86 in β6, and v) N27 in the L1 loop.

## Discussion

When a peptide binds to a domain, dynamical changes occur at the level of the residue side-chains reminiscent of some form of information exchange possibly distributed over the entire domain structure. Experimental work on hPDZ2 has shown that these dynamical effects are indeed present, that manipulating the residues involved shows affinity changes, and that these effects may vary between members of the same domain family. Here we show, through an information theoretical analysis of the dependencies between the side-chain conformations of all domain residues, that a (sub-)network of dynamical changes can be predicted from the publically available structures, showing strong overlap with the experimental data [3]. However, as indicated in the Results section, additional methyl-group bearing residues are identified for which experimental data do not provide confirmation, as is the case of I35 and T77, or for which relaxation data are completely or partially missing (see for instance V37, T28, V75 and the residues in DS3). Nevertheless, the potential dynamical importance of these residues becomes clearer when one also takes into account the dynamical effects produced by the mutants H71Y, I35V and I20F as reported in [18]. Mutating the residues H71 and I20 indicates that both T28 and V37 experience changes in the local correlation times. The marginal role of H71 in hPDZ2 as discussed in [18] is also observable in the network of all dynamic effects visualized in Figure 2. In comparison to I20, H71 has a much less central role to play in the network of dynamical effects.

The I35V mutant, which did not show any dynamical role in [3], not only affects residues located in the BS and DS1 regions, but also induces changes in DS3 residues V9 and L89, which may be linked to I35 via L11 and L87. At this point we can only speculate about why no dynamical changes were observed experimentally for I35: as the NMR relaxation experiments provide one value to describe the aggregate dynamic effect induced on a particular residue like I35, the actual importance of this residue may become masked when tightening (increases in ΔMI) and relaxation (decreases in ΔMI) effects are somehow balanced. As I35 (see Figure 2A) is one of the very few residues that experiences strongly both types of effects, we could hypothesize that its importance has indeed been concealed in this manner. To validate this supposition one needs to examine the influence of both negative and positive weights in the cluster extraction method, which is currently not the case since the absolute ΔMI values are used to extract the informative group of residues.

Hence, next to the already strong overlap with the experimental results in [3], all the additional experimental data show that our predictions provide a rather accurate picture of the intra-domain communication induced by the RA-GEF peptide. Interestingly, the regions most distant from the binding pocket that are identified here (like the DS3 and DS2 regions and the DS2-DS4 linker) lie close to the region that experiences changes as a result of the interaction between the first and second PDZ domains of PTP-BL [7]. At this point its is unclear whether this overlap has any allosteric relevance. Surely, further NMR analysis of the interplay between hPDZ1 and hPDZ2 will allow us to identify the inter-domain network of dynamical changes and shed light on the functional interplay between them.

In addition, other predictive approaches, including the work described in [29] and [21], support the potential relevance of residues for which experimental data are at this point not available. For instance, the DS4 region overlaps strongly with the second signal transduction pathway proposed in [20], while the effects observed in the first helix can be related to the first pathway proposed in the same work. The major difference with our predictions is that Kong and Karplus do not identify any of the core residues that were experimentally identified. Interestingly, the results reported in [20] seem to coincide more closely to those reported in Figure 4, suggesting that their approach focused more on backbone and surface effects rather than on side-chain effects in the core of the domain. Potentially, this also might explain the poor predictive scores of their method (see Table 1), as our analysis was derived on the basis of methyl-relaxation data only.

Although the predictive accuracy of our method is clearly better than those provided by the null models and the other computational methods, our approach also suffers from a specific limitation, as it is unable to predict the dynamical effects of alanine residues from only the side-chain dynamics. The small side-chains of alanine residues are conformationally invariant and thus show a uniform distribution corresponding to a single conformation, which implies no uncertainty associated to the outcome of the corresponding random variable. Consequently, this results in zero entropy values and hence zero mutual information shared with the other residues in the domain. To test the impact of this issue, we also calculated the ROC curves excluding the alanine residues from the quality assessment (Figure 3B). This adjustment improves the predictive quality of our method to AUC values of 0.75 and 0.81 for the analysis of all residues or methyl-group containing residues, respectively. Clearly, these results are also better than those obtained for the betweenness-centrality and the random models (see also Table 1). Interestingly, the performance of the predictor based on the betweenness-centrality computed for the bound form also improves, suggesting that alanine residues A39 and A69, which have shown experimentally significant changes in methyl dynamics, are not very central with respect to the other residues in the domain. In contrast to the previous case, the predictor based on the betweenness-centrality computed for the bound form performs better than the one based on the unbound form. This suggests that A39 and A69 become even less involved in response to peptide binding.

Using the best predictive threshold for the network of dynamics changes in hPDZ2, we also determined the network of dynamical changes for the mouse variant of this domain. We show in the alignment of Figure S6 in Text S1 all the similarities and differences between both domains. Some of the differences in the predictions between mPDZ2 and hPDZ2 are clearly due to sequence differences, because they involve either divergent residues such as K43 (see Figure 5), or residues very close to the mutations (see Figure 5 and Figure S6 in Text S1). Some others, like R79, most probably reflect the ligand-induced conformational change of helix α2 in response to the APC peptide [4], which is not observed for the RA-GEF2 peptide in the hPDZ2 case.

Eight methyl-group bearing residues, four for each domain, characterize the differences between the two variants (see Figure 5): four residues (i.e. V37, V22, V75, T77) oriented orthogonally with respect to the binding groove are predicted for the hPDZ2, while a set of three additional residues (V58, L59, L88) at the opposite side of the binding groove, and I41 located on the β3-α1 linker, are predicted for mPDZ2. The inclusion of the latter residue may simply be a consequence of the sequence differences between hPDZ2 and mPDZ2 in that region (see Figure 5). Furthermore, the differences in peptide between hPDZ2 and mPDZ2, i.e. the RA-GEF2-derived peptide vs. the APC-derived peptide, may easily have induced the differences between hPDZ2 and mPDZ2. In addition, the clustering algorithm requires a threshold *t* to extract the informative group from the matrix of dynamical changes, which was optimized for hPDZ2. Clearly, in order to establish an optimal threshold for *t*, it should have been determined on the basis of a number of independent datasets of different proteins. Currently, we cannot exclude that the value *t* selected here may add or exclude certain residues unwillingly. Nevertheless, our results show clearly that the dynamic regions within the core of both hPDZ2 and mPDZ2 are almost equivalent.

One particular difference between both domains is the amount of positive and negative changes, visualized by red and blue links, respectively, in Figure 2 and Figure S5 in Text S1. In our analysis of hPDZ2, the number of pairs of residues experiencing decreases in the conformational coupling due to binding is significantly lower than those obtained in mPDZ2. It is unclear whether this is simply an artifact of the data or a functionally relevant dynamics. Further experimental analysis of these structures may provide insight into this issue.

Finally, even though for hPDZ2 our approach has a higher accuracy than the other methods, great care must be exercised in generalizing the conclusions since only one complete data set is available. To further evaluate and improve all current methods additional NMR data sets, containing both structural and dynamical information, are required. Only then we can assess if these methods are capable of assisting biological research in understanding the mechanisms of both intra-protein communication and allostery.

## Materials and Methods

### NMR and crystal structure data

The human variant of the PDZ2 bound and unbound forms, used in this paper, correspond to NMR PDB structures 1D5G [30] and 3PDZ [31], and to the PDB crystal structures 3LNY and 3LNX [17], respectively. The mouse variant of the PDZ2 bound and unbound forms correspond respectively to the NMR PDB structures 1VJ6 [4] and 1GM1 [27]. Every structure of an ensemble was first energy minimized using YASARA with the Yamber2 force field [32].

### Sampling the side-chain conformational space

To sample the conformational space of each side-chain, we need to identify a fine-grained alphabet of side-chain conformations. As explained before in detail [26], a statistical rotamer dataset based on conditional statistics of dihedral angles was derived from the WHAT-IF dataset [33] and added to the FoldX software. Details concerning the FoldX force field can be found in [34], [35].

This fine-grained dihedral angles dataset supplies a high-resolution enumeration of the side-chain conformational space for every residue in the structure. This position-specific alphabet covers the conformational space of the residue side-chain over all backbones, ensuring in this manner that all acceptable conformations can be visited during the sampling process. Once the alphabet is determined, we sample the conformational space of every side-chain relative to a particular backbone conformation using Monte-Carlo Sampling with Metropolis Criterion.

Each Monte-Carlo process takes one backbone extracted from the NMR ensemble as input, alters the side-chain conformations towards other favourable states present in the alphabet, and stores at regular intervals (every 1000 iterations) the conformational state of the entire domain. The FoldX force field determines whether a certain change in conformational state is favourable by calculating its free energy change ΔG. The Metropolis criterion states that this change is accepted with a probability *p* as given by the formula: 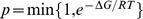. Metropolis Monte Carlo sampling of side-chain conformation was performed at 298K.

Prior to the actual sampling, the process first executes an energy minimization phase, to ensure that the sampling commences from a low-energy state. In order to collect many data points we ran the Monte-Carlo sampling process many times in parallel using the same backbone starting conformation, combining the results into one file when all processes were finished. In our simulations we recorded, for each ensemble, 751500 domain conformations.

### Computing the side-chain mutual information coupling

The sampling procedure is applied to the ensembles in the two states (bound and unbound). Each one of the sets of sampled conformations is composed of side-chain configuration distributions of each residue in the domain. Therefore, each residue corresponds to a random variable, and its realizations correspond to the side-chain conformations the residue takes. The mutual information between two of these distributions quantifies the amount of dependency (or information transfer) between side-chain movements of two residues, details discussed in [26].

Given two discrete random variables *A* and *B*, the mutual information *I(A,B)* is computed as *I(A,B) = H(A)+H(B)−H(A,B)*, where *H(A)* and *H(B)* are the Shannon's entropy and *H(A,B)* is the joint entropy between *A* and *B*, defined as follows:
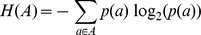


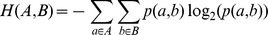



We look at the changes in dynamical couplings upon a binding event by computing the absolute difference in mutual information between the two states: 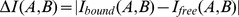. The result is the matrix of absolute changes shown in Figure S1 in Text S1 for hPDZ2.

Since the original method, discussed in [26], a number a new pre-processing steps were introduced on the data to improve the calculation of Shannon's entropy and MI. First, the conformational data for each residue was reparametrized to the unit interval [0,1], which does not change the outcome since MI is invariant to reparametrization of the marginal variables [36]. Yet this step is important since it removes differences between the alphabet sizes of each residue. In addition, we added to each reparametrized value a low amplitude Gaussian noise (mean zero and variance ∼10^−12^), smoothing out the probability distribution. This new data was then discretized using the fixed frequency discretization method [37], which is capable of managing discretization bias and variance, reducing calculation errors in the analysis and hence improving the quality of the MI estimation.

The MI values computed from the sampled distributions, which were close to those expected for the randomized distributions (i.e. close to zero) of each residue pair, were filtered out of the matrix, since they were considered of no relevance for the major information flows throughout the structure.

### Clustering highly coupled residues

Given the matrix of dynamical changes, we applied the Cluster Affinity Search Technique (CAST) [38] for identifying the residues, which are most relevant for the intra-domain communication. The algorithm searches for cliques of coupled residues above a given affinity threshold *t*. Every time a new cluster is created, the algorithm alternates between “add” and “remove” steps until the cluster stabilizes. High affinity residues are added to the current clique and low affinity ones are removed. The affinity of a residue *r_i_* with respect to a clique *C* is defined as 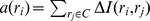, the sum of the changes in mutual information coupling of the residues belonging to the clique. High affinity residues are those for which 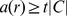. Therefore, by increasing or lowering the affinity threshold one can include more or less residues in the set of predicted ones.

### Network of residue contacts

We built the network of residue contacts starting from the crystal structures of hPDZ2 in the two states. An edge between two vertices (residues) is added if the two residues have at least one pair of atoms at a distance less than or equal to 5 Å in the crystal structure. The resulting networks show small-world properties [39]: they have a clustering coefficient of 0.54 and a characteristic path length of 2.9. The same has been shown for other protein residue networks [40]. Other properties of our networks are a diameter of 6 and an average node degree of 9.9. To build the network of short-range dynamical effects we mapped on the edges the changes in mutual information observed from the unbound to the bound state. The result for hPDZ2 is shown in Figure 2.

The measure of node betweenness-centrality proposed in [41] was selected as the best performing one among a set of betweenness-centrality measures (see Text S1). An extension of this measure was used in [41] to show that the most centrally conserved residues are in agreement with those experimentally predicted to mediate signalling in five different proteins.

### Assessing the quality of the predictions - ROC curves

ROC curves give a picture of the overall performance of a predictor, which is not dependent on the chosen discrimination threshold. We drew ROC curves by varying the threshold *t* and computing False Positive Rate (FPR) and Sensitivity or True Positive Rate (TPR) levels. *FPR* and *TPR* are computed comparing the predictions of a computational approach with the residue labeling based on experimental results reported in [3], in the following way:
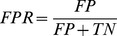


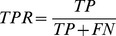
where True Positives (TP) are the residues predicted as relevant by the computational approach that are also relevant from the experimental results, True Negatives (TN) are the residues that are not predicted by the computational approach and have no significant changes in dynamics according to the experimental results, False Positives (FP) are those residues erroneously predicted by the computational approach according to the experimental evidences, and False Negatives (FN) are those residues which are not predicted by the computational approach but have been observed to be relevant in the experimental results.

### Computation of backbone variations

Backbone variations are computed starting from the two NMR ensembles of the domain in the two states. After superposing all structures in the NMR ensemble, by using the MUSTANG structural alignment algorithm [42], we computed, for each residue, the average distance of the C_α_ atoms from their centroid and we took it as a measure of backbone flexibility. We then calculated the differences in flexibility between the bound and the unbound state. We computed z-scores of these differences and we highlighted, in Figure 4, only the most significant ones (the four flexible N-terminal amino acids and the four flexible C-terminal amino acids were not included in this calculation).

### Combining side-chain dynamics and backbone information

The results of the two predictors, one based on the change in MI shared between residue side-chains and the other based on the change in backbone flexibility, were combined by taking the two ranks of the residues, in the following way:
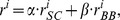
where 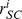 is the position of residue *i* in the ranking produced by our side-chain dynamics based predictor, and 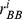 is the position of the same residue in the ranking produced by the backbone variation based predictor. *r^i^* results from the weighted sum of the ranking positions. The final rank of predictions is obtained by sorting the residues according to the resulting *r^i^*, while breaking ties at random. The ROC curve of the resulting predictor is reported in Figure S3 in Text S1. We fixed *α* = 1.5 and *β* = 1 by giving a 50% more importance to the side-chain dynamics information with respect to the backbone one.

## Supporting Information

Text S1 Supporting information: Accurate prediction of the dynamical changes within the second PDZ domain of PTP1e. The text contains additional information, figures and tables related to the manuscript.
(PDF)
