# Supplementary material for: Accurate Prediction of the Dynamical Changes within the Second PDZ Domain of PTP1e
Source: PLoS Comput Biol. 2012 Nov 29;8(11):e1002794. doi: 10.1371/journal.pcbi.1002794 (PMC3510070; doi:10.1371/journal.pcbi.1002794)
Supplement: Text S1 — Supporting information: Accurate prediction of the dynamical changes within the second PDZ domain of PTP1e. The text contains additional information, figures and tables related to the manuscript. (PDF) [file pcbi.1002794.s001.pdf]

Supporting Information:

Accurate prediction of the dynamical changes within the second PDZ domain of PTP1e

Elisa Cilia[1], Geerten W. Vuister[2] and Tom Lenaerts[1,3]

[1] – MLG, Département d'Informatique, Université Libre de Bruxelles, Boulevard du Triomphe CP212, 1050 Brussels, Belgium

[2] – Department of Biochemistry, University of Leicester, Henry Wellcome Building, Lancaster Road, Leicester LE1 9HN, United Kingdom

[3] – AI-lab, Vakgroep Computerwetenschappen, Vrije Universiteit Brussel, Pleinlaan 2, 1050 Brussels, Belgium

Measures of betweenness centrality

We evaluated different measures of shortest path betweenness centrality. We computed node and edge betweenness centrality measures proposed in [1] and [2]. In [1], the betweenness centrality of a vertex or edge k is given by:

$$c_B = \sum_{i,j \in V} \frac{\sigma(i,j|k)}{\sigma(i,j)}, \quad \text{(Eq. 1)}$$

where V is the set of vertices (residues) in the network, σ counts the number of shortest paths between vertices i and j, and $\sigma(i,j|k)$ counts the number of shortest paths passing trough vertex/edge k [3,4]. We normalized this measure over the number of vertices/edges in the network. The measure proposed in [2] is slightly different. It corresponds to the change in the characteristic path length (L) observed by removing from the network a certain vertex or edge k:

$$\Delta L_k = |L - L_{rem,k}| \quad \text{(Eq. 2)}$$

The characteristic path length L is essentially the average shortest path length $\frac{1}{N}\sum_{j>i} d(i,j)$, where N is the total number of vertex pairs and $d(i,j)$ is the shortest path length between two vertices i and j.

We also computed node betweenness centrality as proposed in [5]. In that case the algorithm works by removing at each step the vertex with highest betweenness-centrality (according to Eq. 1) and its incident edges. Then, it computes again the betweenness centrality values for the residues affected by the removal in the remaining network. This is repeated until no more edges remain, or the network splits into different connected components.

For extracting the most relevant residues from the edge betweenness centrality matrix we applied again the CAST algorithm. Edge betweenness centrality measures resulted in a less discriminant power in predicting allostery-involved

residues in hPDZ2 with respect to node betweenness centrality ones (data not shown). We selected the measure of node betweenness centrality in Eq. 2 as the best performing one in this context and we compared to that (see Figure S2 and Table S2).

Note that, we have also looked at the performance of a predictor based on the changes in betweenness centrality happening upon binding the peptide. Still, these measures of centrality gave us better results when directly computed on the networks built from the ligand-free or ligand-bound structures (see Figure S2 and Table S2).

Tables

Residues	Labeling	Residues	Labeling
**ILEA6**	*N*	**VALA58**	*N*
**VALA9**	*M*	**LEUA59**	*N*
**LEUA11**	*M*	**ALAA60**	*M*
**ALAA12**	*M*	**VALA61**	*P*
**LEUA18**	*P*	**VALA64**	*P*
**ILEA20**	*P*	**LEUA66**	*P*
**VALA22**	*P*	**ALAA69**	*P*
**THRA23**	*N*	**THRA70**	*N*
**VALA26**	*P*	**ALAA74**	*M*
**THRA28**	*M*	**VALA75**	*M*
**VALA30**	*P*	**THRA77**	*N*
**ILEA35**	*N*	**LEUA78**	*P*
**VALA37**	*M*	**THRA81**	*P*
**ALAA39**	*P*	**VALA84**	*M*
**VALA40**	*P*	**VALA85**	*P*
**ILEA41**	*N*	**LEUA87**	*M*
**ALAA45**	*N*	**LEUA88**	*M*
**ALAA46**	*N*	**LEUA89**	*M*
**ILEA52**	*N*	**THRA96**	*M*

Table S1. Assignment of classes to the experimentally derived hPDZ2 residues. The methyl side-chain residues are labelled as positive (*P*), negative (*N*), or missing (*M*) according to the experimental results reported in [6]. Positive residues are those for which NMR methyl side-chain relaxation experiments showed a significant change in S^2 and τ_e parameters (the difference observed in the fitted parameter was twice the error [6]. On the opposite, negative residues are those for which the change is not significant according to the same criterion. Finally, residues for which there are missing or contradictory data, and due to this uncertainty cannot be included in the positive or negative sets, are

considered as missing values and therefore not included in the computation. Note that V9 is included in the missing data because subsequent results reported in [7] have shown a different dynamics response of this residue to key mutations like I35V and I20F and its response to peptide binding.

NBC Predictor	AUC (bound)	AUC (unbound)	AUC (changes)
Freiman betweenness centrality	0.49	0.5	0.44
Del Sol betweenness centrality	0.59	0.55	0.55
Newman betweenness centrality	0.5	0.34	-

Table S2. Areas Under the ROC Curves (AUC) shown in Figure S2, for the Node Betweenness Centrality (NBC)-based predictors.

t	Predictions using only METHYL residue changes (Figure 1A)	*t*	Predictions using ALL residue changes (Figure S1)
0.193	L87, L89, V9, L11, **V26**	0.189	L87, L89, V9, H71, D5, Y36, L11, **V26**
0.101	+ **I20**	0.120	+ R57, **L78**
0.094	+ **L78**	0.115	+ **L66**
0.081	+ **V40**	0.086	+ S29, D56, H53, **V30**
0.076	+ **V30**	0.085	+ *T77*
0.063	+ V37, V75, *T77*	0.083	+ **V40**
0.054	+ **L66**	0.081	+ *I35*
0.053	+ *I35*	0.071	+ E47, **I20**
0.048	+ **L18**	0.067	+ **T81**
0.045	+ **T81**	0.043	+ K54, R51, V75, K13, F7, E67, H86, V37, **L18**
0.034	+ **V22**	**t_2=0.027**	+ S17, S21, D49, N27, T28, **V22**
0.026	+ **V61**	0.025	+ R31, T70
**t_1=0.023**	+ T28, **V85**	0.024	+ I52
0.021	+ L88, T70	0.022	+ N16, L88, **V85**
0.02	+ I52	0.021	+ **V61**
0.018	+ V58	0.019	+ H32, E8, T23
0.016	+ T23	0.017	+ V58
0.015	+ I41	0.015	+ D15, S48, I41
0.012	+ T96, L59	0.013	+ Q93, L59
0.007	+ V84, I6	0.009	+ K91, E10, T96, V84, I6
0.006	+ **V64**	0.007	+ E90, K72, S94, K2, **V64**
0	+ A12, **A39**, A45, A46, A60, **A69**, A74	0	+ P3, N14, N62, S65, E76, R79, N80, P1, K38, Q43, Q73, P42, Q83, P95, G4, A12, G19, G24, G25, G33, G34, **A39** + G4, A12, G19, G24, G25, G33, G34, **A39,** G44, A45, A46, G50, G55, A60, G63, G68, **A69**, A74, G82, G92

Table S3. Predictions corresponding to the different points of the ROC curves in Figure 3A, by decreasing the threshold *t* of the clustering algorithm. The bold lines indicate the points in the ROC curves corresponding to the best performing predictors. The experimentally identified residues are annotated in bold.

Figures

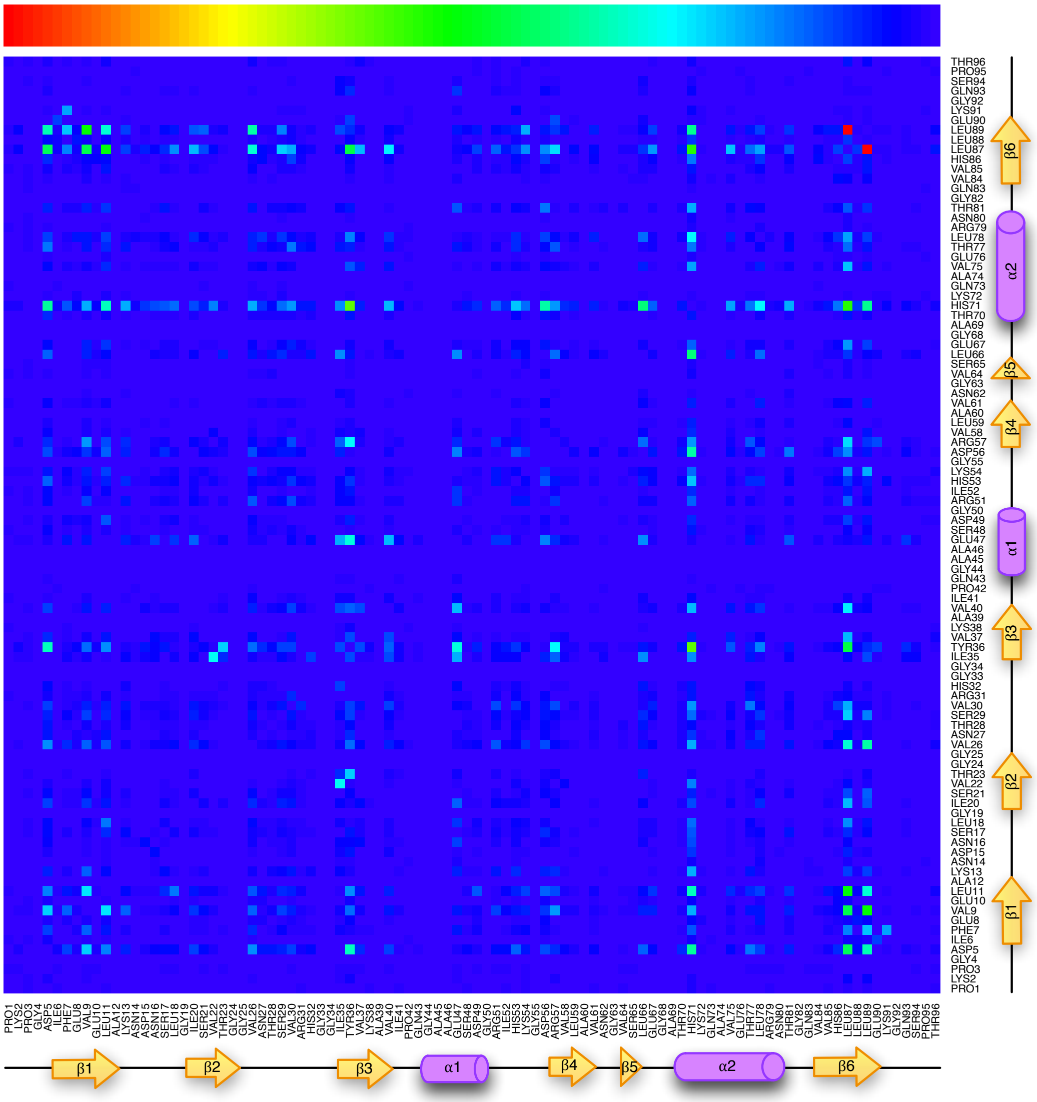

Figure S1. A representation of the matrix of dynamical changes (heat map) in hPDZ2. Absolute differences in side-chain coupling, normalized between 0 and 1, are represented with colors from blue (0) to red (1) according to the color scale reported on top. Given two residues R_1 and R_2, it corresponds to $|\boldsymbol{I}_{\boldsymbol{bound}}(\boldsymbol{R}_1, \boldsymbol{R}_2) - \boldsymbol{I}_{\boldsymbol{free}}(\boldsymbol{R}_1, \boldsymbol{R}_2)|$, i.e. the absolute difference of mutual information between the residues side-chain conformational distributions in the bound and unbound states (see also Materials and Methods). The amino acid sequence is annotated with the corresponding secondary structure elements.

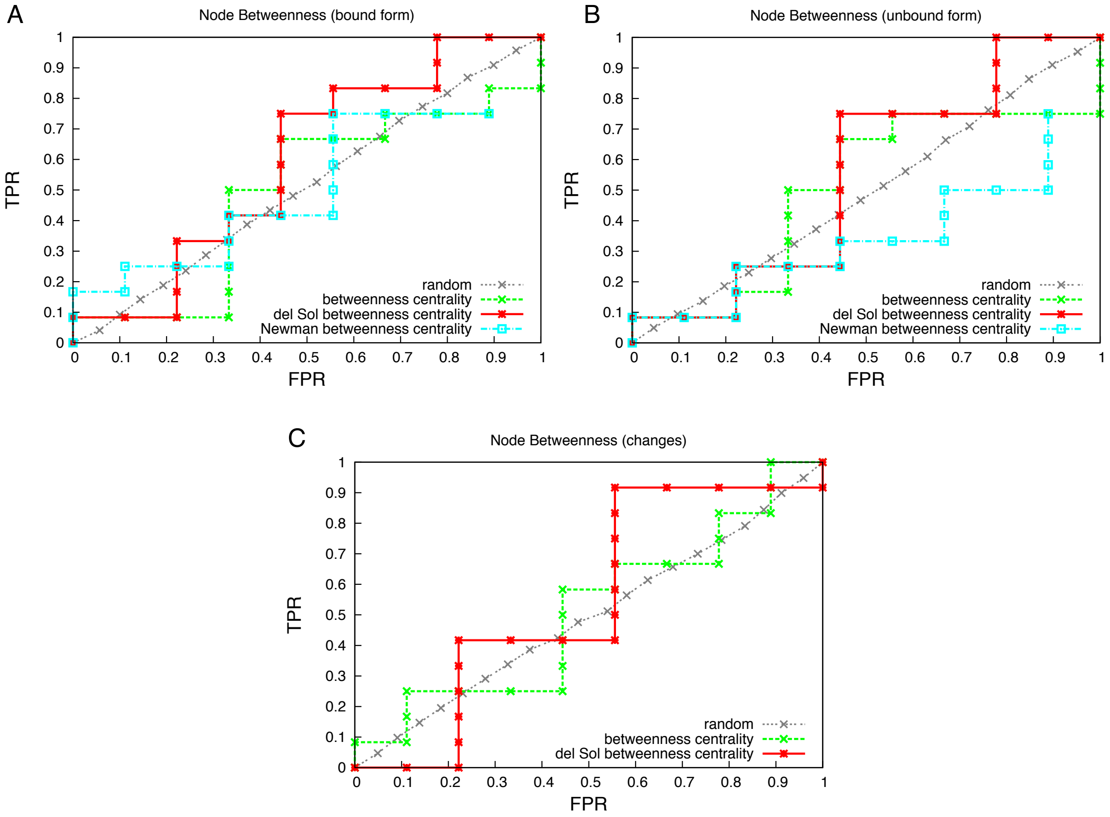

Figure S2. ROC curves of the node betweenness centrality-based predictors. A) Node betweenness centrality is computed according to different algorithms [1], [2], [5], starting from the structure of hPDZ2 in the bound form. B) Node betweenness centrality is computed starting from the structure of hPDZ2 in the unbound form. C) Residues are ranked according to the difference in their node betweenness centrality in the two states (bound and unbound).

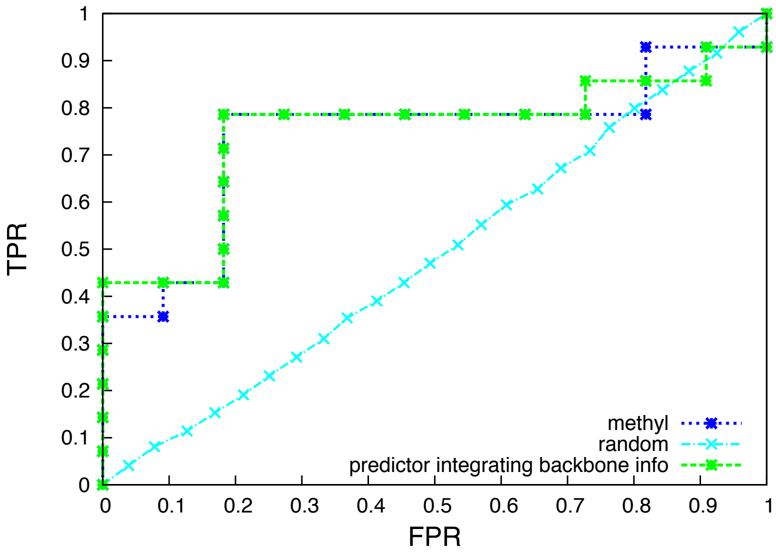

Figure S3. ROC curve for the predictor based on integrated information extracted from backbone variations and side-chain dynamics. A very slight AUC improvement can be noticed (from 0.74 to 0.75).

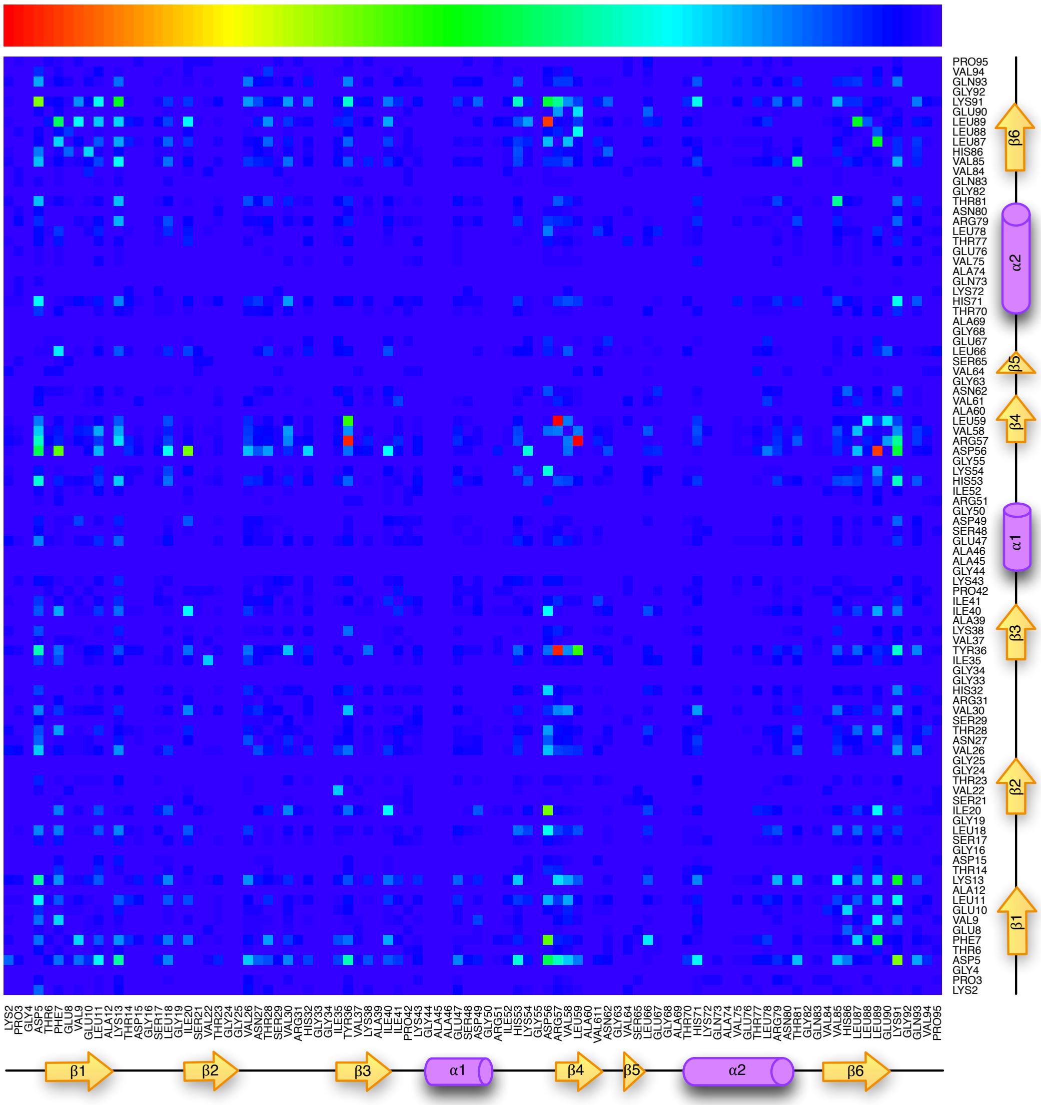

Figure S4. Heat map of the matrix of dynamical changes computed for mPDZ2. The numbering of the amino acid is referred to the alignment in Figure 1C. The amino acid sequence is annotated with the corresponding secondary structure elements.

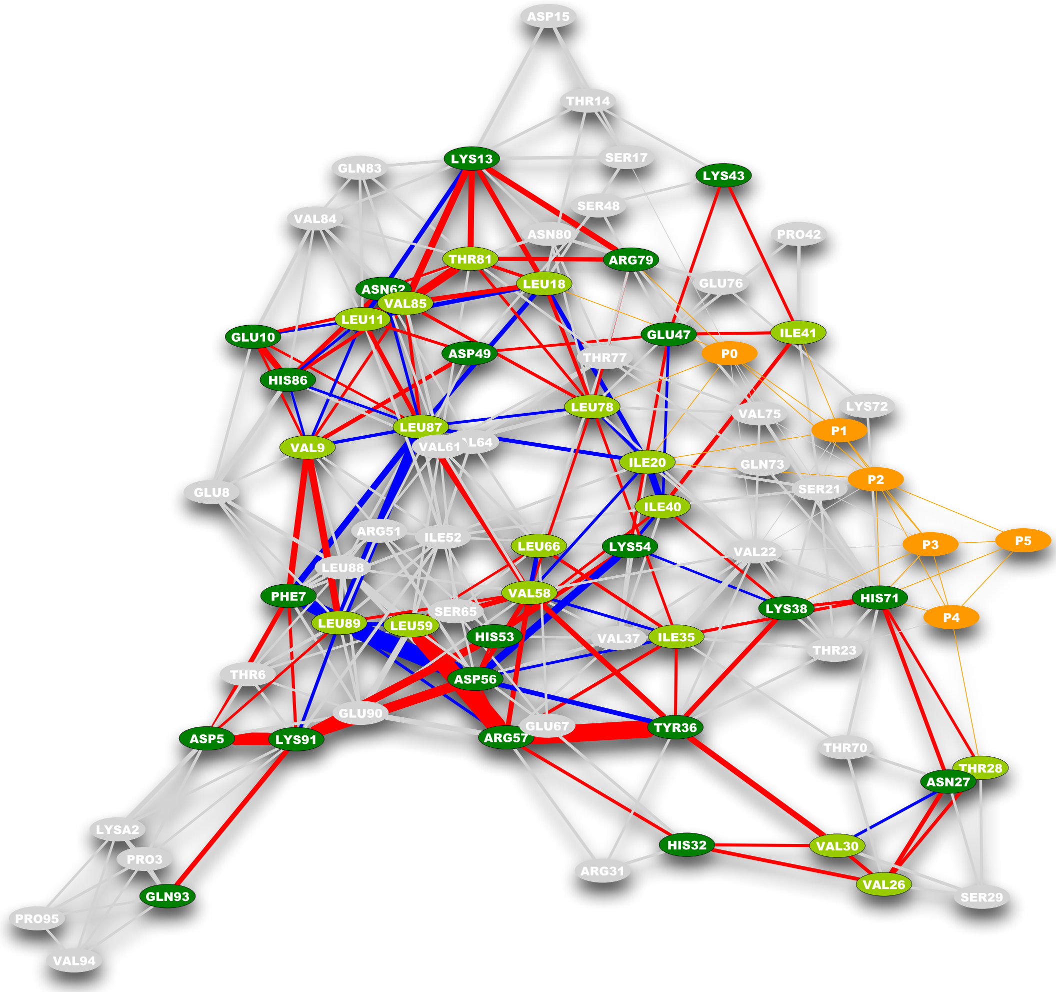

Figure S5. Network of the short-range dynamical changes in mPDZ2. Residues highlighted in green are those predicted from the complete matrix of changes in MI coupling (light green for the methyl side-chain bearing ones and dark green for the others). Red edges represent an increase in MI coupling upon the binding event, while blue edges represent a decrease in coupling. The thickness of the edges represents the amount of the change. Peptide residues and their contacts with the domain residues are highlighted in orange. The network visualization follows the organic layout implemented in Cytoscape [8]. Note that, for mPDZ2 the network of residue contacts is extracted from the NMR ensembles by including only the contacts present in more than the 50% of the backbones of the ensemble.

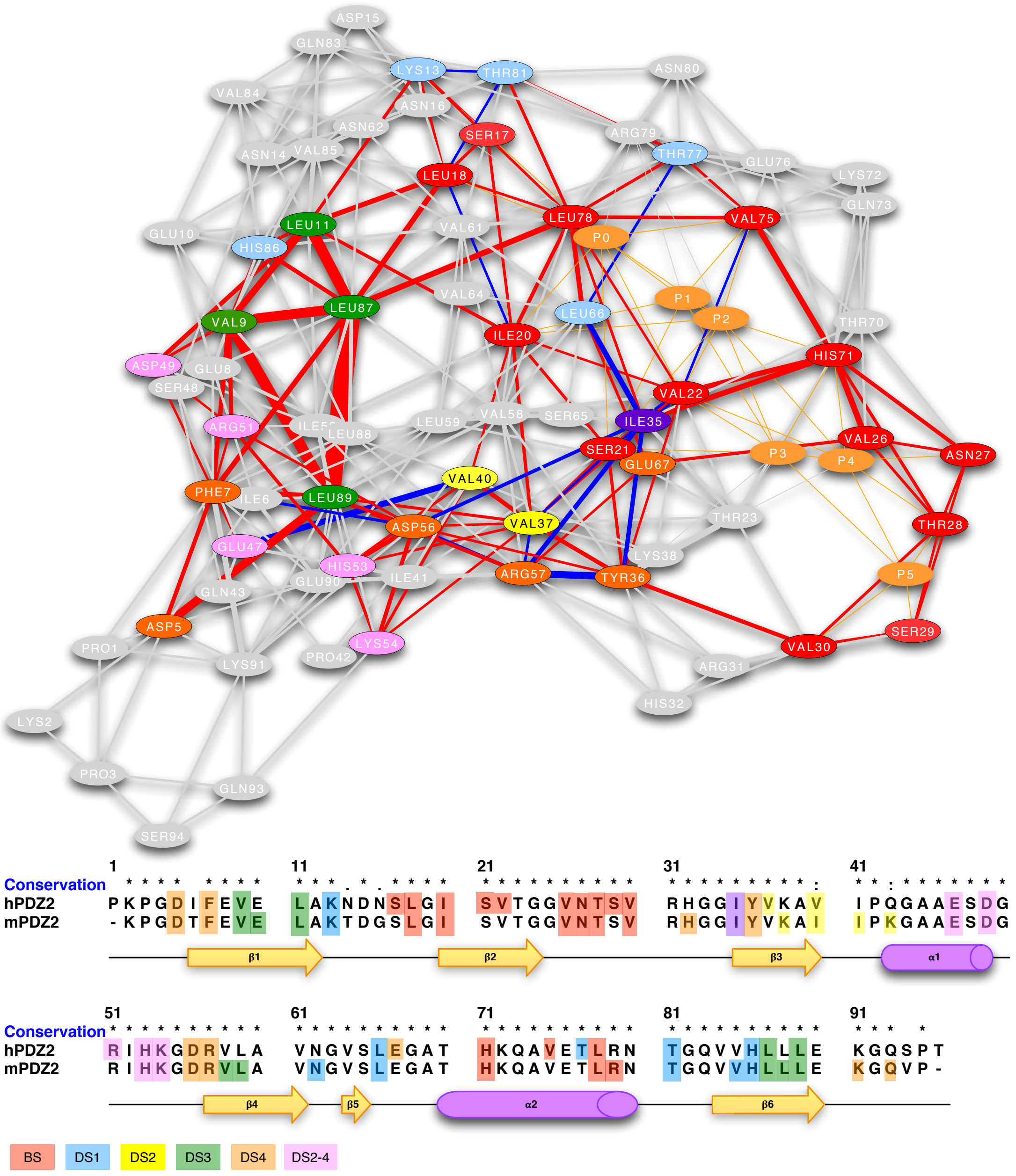

Figure S6. The predictions based on mutual information couplings from the matrices in Figure S1 and S4 are mapped in a sequence alignment of the two homologous domains (hPDZ2 and mPDZ2), and on the network of changes in mutual information for hPDZ2. The regions discussed in the paper are highlighted with different colors (binding site (BS), distal region 1, 2, 3 and 4 (DS1, DS2, DS3, DS4) and the linker region between DS2 and DS4 (DS2-4) also shown in Figure 2C. The alignment also reports the secondary structure of the domains.

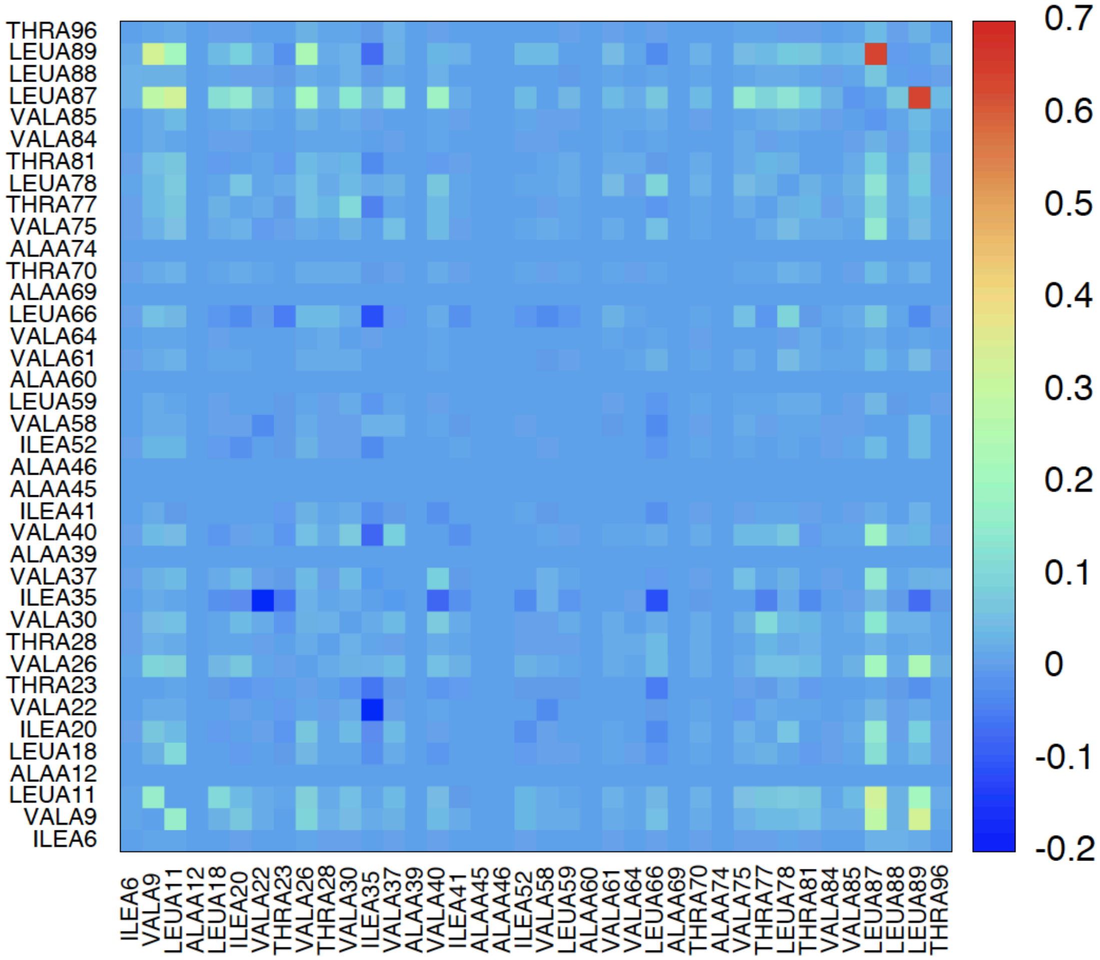

Figure S7. The matrix of raw ΔMI values for the methyl-group bearing residues. The matrix colors the values from blue (-0.2) to red (0.7) according to the color scale reported on the right. To produce the matrix in Figure 1A, the absolute values were taken of each ΔMI and then these absolute values were normalized between 0 and 1.

References

1. Freeman LC (1977) A set of measures of centrality based on betweenness. Sociometry 40: 35-41.

2. del Sol A, Fujihashi H, Amoros D, Nussinov R (2006) Residues crucial for maintaining short paths in network communication mediate signaling in proteins. Molecular systems biology 2: 2006 0019.

3. Brandes U (2001) A faster algorithm for betweenness centrality. Journal of Mathematical Sociology 25: 163-177.

4. Brandes U (2008) On variants of shortest-path betweenness centrality and their generic computation. Social Networks 30: 136-145.

5. Girvan M, Newman MEJ (2002) Community structure in social and biological networks. Proceedings of the National Academy of Sciences of the United States of America 99: 7821-7826.

6. Fuentes EJ, Der CJ, Lee AL (2004) Ligand-dependent dynamics and intramolecular signaling in a PDZ domain. Journal of molecular biology 335: 1105-1115.

7. Fuentes EJ, Gilmore SA, Mauldin RV, Lee AL (2006) Evaluation of energetic and dynamic coupling networks in a PDZ domain protein. Journal of molecular biology 364: 337-351.

8. Smoot ME, Ono K, Ruscheinski J, Wang PL, Ideker T (2011) Cytoscape 2.8: new features for data integration and network visualization. Bioinformatics 27: 431-432.
